# Genome Analysis of a Novel Clade b *Betabaculovirus* Isolated from the Legume Pest *Matsumuraeses phaseoli* (Lepidoptera: Tortricidae)

**DOI:** 10.3390/v12101068

**Published:** 2020-09-23

**Authors:** Ruihao Shu, Qian Meng, Lin Miao, Hongbin Liang, Jun Chen, Yuan Xu, Luqiang Cheng, Wenyi Jin, Qilian Qin, Huan Zhang

**Affiliations:** 1 State Key Laboratory of Integrated Management of Pest Insects and Rodents, Institute of Zoology, Chinese Academy of Sciences, Beijing 100101, China; shuruihao@ioz.ac.cn (R.S.); mengqian@ioz.ac.cn (Q.M.); miaolin@ioz.ac.cn (L.M.); chenglq1995@gmail.com (L.C.); jwy707508523@126.com (W.J.); qinql@ioz.ac.cn (Q.Q.); 2 National Animal Collection Resource Center, Institute of Zoology, Chinese Academy of Sciences, Beijing 100101, China; lianghb@ioz.ac.cn (H.L.); chenj@ioz.ac.cn (J.C.); brjdgryx@163.com (Y.X.); 3 College of Horticulture and Plant Protection, Yangzhou University, Yangzhou 225009, China; 4 College of Life Sciences, Hebei University, Baoding 071002, China

**Keywords:** granulovirus, baculovirus genome, biological control, pest management

## Abstract

*Matsumuraeses phaseoli* is a Lepidopteran pest that primarily feeds on numerous species of cultivated legumes, such as *Glycine* and *Phaseolus*. It is widely distributed in northeast Asia. A novel granulovirus, designated as *Matsumuraeses phaseoli granulovirus* (MaphGV), was isolated from pathogenic *M. phaseoli* larvae that dwell in rolled leaves of *Astragalus membranaceus*, a Chinese medicinal herb. In this study, using next-generation sequencing, we report the complete genome of MaphGV. MaphGV genome comprises a double-stranded DNA of 116,875 bp, with 37.18% GC content. It has 128 hypothetical open reading frames (ORFs). Among them, 38 are baculovirus core genes, 18 are lepidopteran baculovirus conserved genes, and 5 are unique to *Baculoviridae*. MaphGV has one baculovirus repeat ORF (*bro*) and three inhibitors of apoptosis proteins (*iap*), including a newfound *iap-6.* We found two atypical baculoviral homologous regions (*hrs*) and four direct repeats (*drs*) in the MaphGV genome. Based on phylogenetic analysis, MaphGV belongs to Clade b of *Betabaculovirus* and is closely related to *Cydia pomonella* *granulovirus* (CpGV) and *Cryptophlebia leucotreta* *granulovirus* (CrleGV). This novel baculovirus discovery and sequencing are invaluable in understanding the evolution of baculovirus and MaphGV may be a potential biocontrol agent against the bean ravaging pest.

## 1. Introduction

Baculoviruses are double-strand DNA viruses that specifically infect the larvae of insect orders such as Lepidoptera, Hymenoptera, and Diptera [1]. The family *Baculoviridae* is divided into four genera. There is much less genomic information for *Beta*-, *Gamma*-, and *Deltabaculovirus* than for *Alphabaculovirus*. The occlusion bodies (OBs) of *Betabaculovirus* are ovocylindrical structures, generally 0.12 × 0.50 µm in diameter, and are significantly smaller than those of *Alphabaculovirus* (0.15 to 5 µm) [2]. The genus was initially called granuloviruses (GVs) due to the granule-like morphology of the OBs [3]. Each virion of occlusion-derived virus (ODV) typically contains a single nucleocapsid within a single envelope. Baculoviruses typically have narrow host ranges, often limited to just one or a few related insect species [4] and have ancient coevolutionary interactions with their hosts [5]. GVs are confined to the Lepidoptera and are reported to infect 148 hosts in this family [6], much less than members of the Lepidoptera family [7]. This suggests that numerous betabaculoviruses are yet to be discovered.

*Matsumuraeses phaseoli* (Lepidoptera: Tortricidae) is a lepidopteran pest that feeds on cultivated legumes, such as *Glycine* and *Phaseolus*. It characteristically curls up and feeds on the leaves of the plant [8]. The host insect is widespread across northeast Asian countries, such as Japan, North Korea, Russia, and China [9]. In China, the species is an important pest for 11 species of economically important leguminous crops, including vetch, broad bean, alfalfa, and clover [10]. In this study, eight *M. phaseoli* larvae presenting symptoms of baculovirus infection were collected from the glued leaves of milkvetch, *Astragalus membranaceus*. *A. membranaceus* is a perennial legume widely cultivated in China; its roots are exploited for traditional Chinese herbal medicine. In recent years, protracted cultivation has resulted in the unprecedented emergence and occurrence of pests. The use of selective pesticides, such as baculovirus-based products, is particularly important to avoid pesticide residues in food crops and plant-based traditional medicines. Baculoviruses infect and kill insect pests in the field. However, most baculoviruses are host species-specific; therefore, novel discovery and sequencing of viruses specific to insect pests are important. Although granuloviruses pathogenic to *M. phaseoli* have been reported in the past decades [11,12], no research has been documented.

In this study, several *M. phaseoli* larvae presenting symptoms of baculovirus infection were collected from glued leaves of *A. membranaceus*. After characterization, we observed the complete genome of a novel granulovirus isolated from *M. phaseoli* larva with symptoms of baculovirus infection. The host species was identified using morphological characteristics and mitochondrial cytochrome c oxidase I (COI) barcode sequences. Therefore, the putative granulovirus was designated as *Matsumuraeses phaseoli granulovirus* (MaphGV).

## 2. Materials and Methods

### 2.1. Virus Collection and Host Identification

Eight host insect cadavers displaying symptoms of baculovirus infection were directly collected from *A. membranaceus* leaves in northwest China (Min County, Dingxi city, Gansu province) in September 2019. The bodies of the host insects containing the virus were directly extracted using the phenol-chloroform method. The target mitochondrial COI barcode was amplified by polymerase chain reaction (PCR) using lepidopteran COI primers (Table S1) [13]. The amplified fragments were Sanger sequenced and analyzed by BLASTn [14] with the barcode sequence in BOLD [15] and GenBank [16].

### 2.2. Morphological Characterization of the Occlusion Bodies

Typical granulovirus features were observed under the light microscope (Leica DM2000, Leica, Wetzlar, Germany). Pure OBs were obtained after several differential centrifugations. A suspension of the OBs was prepared [17] and detected using a scanning electron microscopy (SEM) (Hitachi SU8010, Hitachi, Tokyo, Japan) at a 5 kV acceleration voltage [18]. The size of the MaphGV OB was measured based on 44 complete image samples captured by SEM.

### 2.3. Genomic DNA Sequencing and Sequence Analysis

Viral genomic DNA was extracted after cleavage of purified OBs in an alkaline solution [19]. The DNA was sequenced using the Illumina Hiseq X system. The high-quality pair-end reads were assembled de novo into contigs using the Iterative Virus Assembler (IVA, version 1.8) [20]. To validate two regions with low mapping rate, PCR amplification, molecular cloning, and subsequent Sanger sequencing were performed. The primers used are listed in Table S1. The strategy allowed for confirmation and correction of the sequences, including the addition of two nucleotides and the elimination of the other six [19]. The genetic variability of the viral isolate was investigated using a bacsnp pipeline [21].

FGENESV [22] and VIGOR [23] were used to predict the hypothetical open reading frames (ORFs) of the MaphGV genome, with at least 50 codons and minimal overlap. The complete genome and annotation information for MaphGV were submitted to GenBank under accession number MT844067. EMBOSS stretcher [24] was used to compare the MaphGV genome with that of related species. Gene parity plots were drawn to assess the pairwise ORF synteny between MaphGV and the selected baculoviruses. [25].

### 2.4. Non-coding Region Analysis

Tandem Repeats Finder [26], REPuter [27], Blast2seq [28], and EMBOSS palindrome [29] were used to search the homologous repeat regions (*hrs*) and direct repeats (*drs*) in the MaphGV genome. The secondary structures of repeat sequences were predicted using the ViennaRNA secondary structure server [30]. The structures of *hrs* were visualized by the IBS online server [31]. The alignment of *hrs* repeat units was visualized using BOXSHADE [32].

To screen early and late promoter motifs, we checked the 180 nt upstream region of each initiation codon. Two potential TATA-box elements were derived by SeqKit [33] for early promoters. One had a common TATA-box motif (TATAW) with a CAKT mRNA start site sequence of 25–35 nt downstream [34,35]. The other was derived from TATA-like elements (TAATWAA) initially found upstream of some *lef* ORFs [36]. DTAAG was treated as a baculovirus late promoter element located, on average, −60 nt upstream of the ORF initiation codon [37].

### 2.5. Phylogenetic Analysis

Protein sequences of 38 baculovirus core genes were extracted from 108 sequenced baculovirus genomes (including MaphGV, Table S2). The sequences were aligned by MAFFT [38] with default parameters. Poorly aligned sites were removed using BMGE [39]. The aligned sequences were then concatenated in the same order as those in the *Autographa californica multiple nucleopolyhedrovirus* (AcMNPV) genome. The phylogenetic relationship was constructed using MEGA version X, based on the minimum evolution method [40].

Kimura two-parameter (K2P) distances [41] were calculated separately for the *granulin*, *lef*-*8*, and *lef*-*9* alignments using the nucleotide distance calculation function of R package “ape” (version 5.3) [42]. Substation rates among sites were set to be uniform. Gaps within the alignment were treated as pairwise deletions.

IQ-TREE (Version 1.6.1) [43] was used to construct the phylogenetic tree for the inhibitor of apoptosis protein (*iap*), based on the maximum likelihood method. The selected substitution model was LG + F + R10.

## 3. Results and Discussion

### 3.1. The Host Determination and Virus Characterization

**Figure 1:**
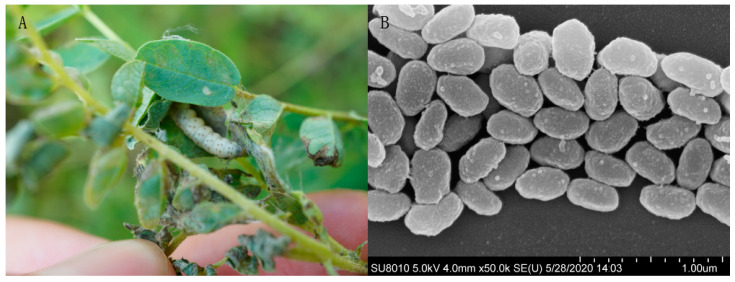
Host larva displaying symptoms of MaphGV infection and the corresponding occlusion bodies. (**A**) *Matsumuraeses phaseoli* larva in the glued *Astragalus membranaceus* leaves exhibiting symptoms for baculovirus infection. (**B**) MaphGV occlusion bodies as seen under scanning electron micrograph. The scale of measurement is marked in the bottom right corner.

The host insects were classified in *Matsumuraeses* genus (Lepidoptera: Tortricidae) according to morphology and damage symptoms of the leaf rolling of the larva. The symptomatic larva presenting pathological features of granulovirus infection is shown in Figure 1A. The sequenced mitochondrial COI barcoding (GenBank accession number: MT578848.1) confirmed the host to be *M. phaseoli*. Based on historical tradition, baculoviruses were named according to the host species from which they were first isolated, followed by their OB morphology group [44]. Accordingly, the virus was named *Matsumuraeses phaseoli granulovirus* (MaphGV).

Ultrastructural analysis revealed that MaphGV OBs are ovocylindrical in shape, a characteristic typical of betabaculovirus occlusion bodies (Figure 1B). They were approximately (395 ± 20) × (239 ± 24) nm, which is within the range of dimensions previously reported for *Betabaculovirus* OBs [44].

### 3.2. Genome Feature

**Figure 2:**
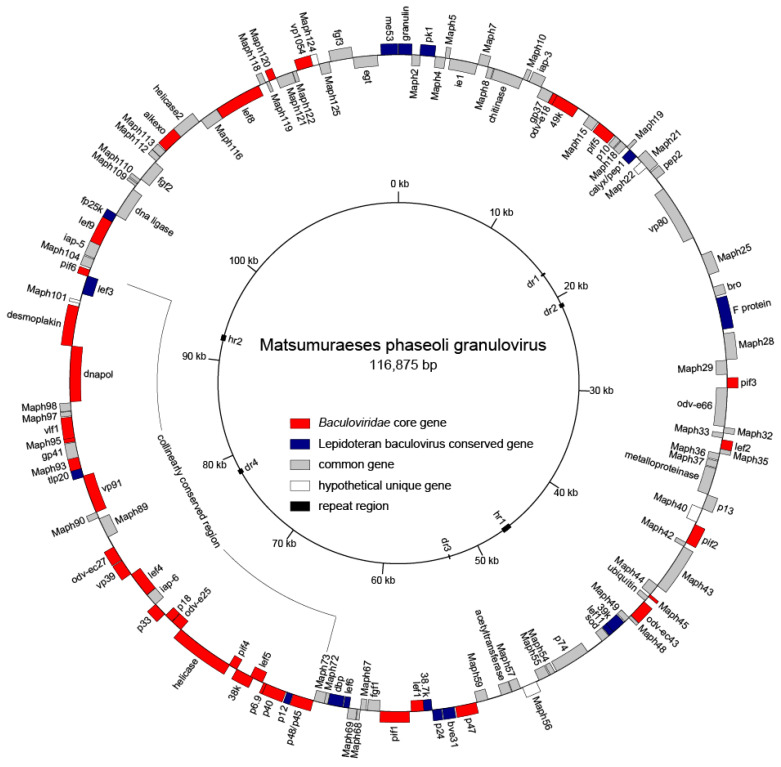
Whole-genome map for MaphGV. Genes in the positive strand are drawn to the outside of the circle and those in the negative strand are on the inside. The gene types are coded as follows: red for core genes, blue for lepidopteran conserved genes, white for hypothetical unique genes, and grey for other common baculovirus genes. Black squares in the inner circle represent repeat regions. The arc indicates the collinearly conserved region.

Using the Illumina Hiseq X sequencing system, 4,868,861 high-quality pair-end reads of MaphGV samples were generated. The complete genome of MaphGV was assembled using IVA (version 1.8). The ambiguous regions were further validated by PCR and Sanger sequencing. Only 11 potential single nucleotide polymorphism (SNP) sites were identified, indicating high purity and low genetic diversity of the isolate (Figure S1). The final MaphGV genome consists of 116,875 bp with 128 hypothetical ORFs (Figure 2). The coding regions cover 89.61% of the genome. The gene encoding granulin was designed as the first ORF, with its start codon designed as the first three nucleotides of the genome. MaphGV contains 38 core genes conserved in all baculoviruses, 16 lepidopteran baculovirus conserved genes [45], and 5 hypothetical unique genes without homologs in *Baculoviridae* (Table 1).

Among the 128 hypothetical ORFs, 123 have homologs in other baculoviruses, including 30 related to virus structure and assembly, 12 to DNA replication, 8 to transcription, 10 to oral infection, and 14 auxiliary genes (Table 1). MaphGV shares 112 homologous ORFs with *Pieris rapae granulovirus* (PrGV), 109 with *Cydia pomonella granulovirus* (CpGV), 105 with *Cryptophlebia leucotreta granulovirus* (CrleGV), 104 with *Choristoneura occidentalis granulovirus* (ChocGV), 95 with DisaGV, 56 with AcMNPV, 35 with NeseNPV, and 17 with CuniNPV. No *cathepsin*, *enhancin*, or *p35* genes were found.

As for the five unique genes, no homolog in GenBank was found through BLASTp search for the following: *orf22* (144 aa), *orf40* (276 aa), *orf56* (280 aa), *orf101* (55 aa), and *orf124* (117 aa). We screened the regions 180 nt upstream of these putative ORFs for promoter elements. The CAKT motif is an initiator element that was found approximately 30 nt downstream away from the TATA-box (RNA-polymerase-II-binding site) at the transcription start site of many genes [34]. *Orf40* harbors a conserved early promoter pattern (TATA-box with the CAKT motif 25-nt downstream), a lef-TATA-like early promoter motif (TAATWAA) [36] and a late promoter (DTAAG) [37]. Both *orf22* and *orf56* possess late promoters [37]. The former harbors a late promoter proximal to the initiation codon (within 15 nucleotides). *Orf101* and *orf124* did not match the elements mentioned above. Further studies are required to explore whether these are functional in MaphGV.

### 3.3. Phylogenetic Analysis of MaphGV

**Figure 3:**
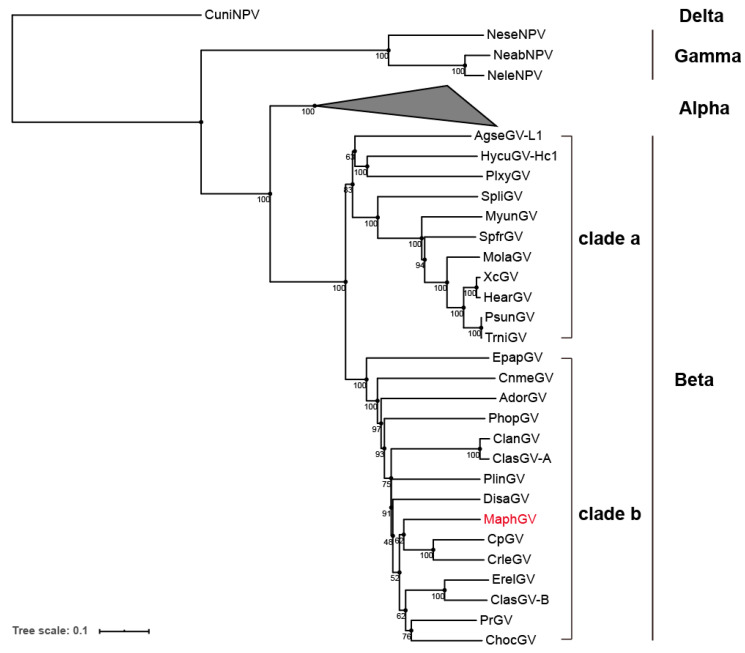
Phylogenetic tree inferred from concatenated predicted amino acid sequences of 38 conserved core genes of 108 sequenced baculovirus genomes. The unrooted tree was generated using the minimum evolution method with 1000 bootstrap values. MaphGV is highlighted in red. Branches of the genera *Alphabaculovirus* are collapsed. Taxon genera are indicated behind corresponding branches. Both clades a and b of *Betabaculovirus* are indicated in brackets.

A phylogenetic tree was constructed based on 38 concatenated predicted amino acid sequences of core genes from 108 completely sequenced baculoviral genomes (including MaphGV). Based on the phylogenetic tree, MaphGV was grouped in clade b of *betabaculovirus* (Figure 3). It is a sister species related to the cluster formed by CpGV and CrleGV, which are isolated from Tortricidae, with 62% bootstrap support. Identification into baculovirus species was based on pairwise nucleotide distances estimated using the K2P model of nucleotide substitution for *polyhedrin*/*granulin, lef-8*, and *lef-9* genes [46,47]. The K2P pairwise distances of the above genes for MaphGV to other granuloviruses were more than 0.05 substitutions/site (Table S3), effectively validating the classification of MaphGV as a novel betabaculovirus species.

**Figure 4:**
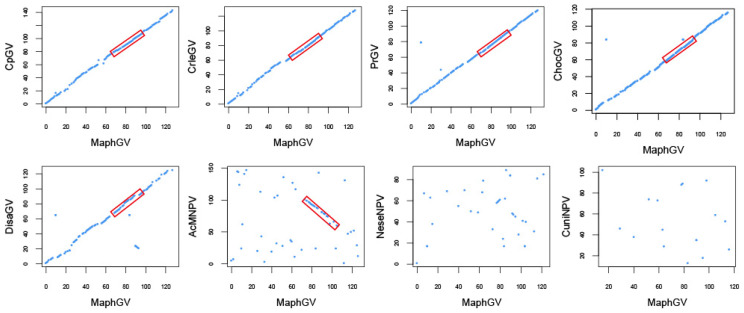
Gene parity plots comparing MaphGV with other baculoviruses. The ORF content and order of the MaphGV genome (x-axis) was compared with that of CpGV, CrleGV, PrGV, ChocGV, DisaGV, AcMNPV (*Alphabaculovirus*), NeseNPV (*Gammabaculovirus*), and CuniNPV (*Deltabaculovirous*) (y-axes). Each point in the plot represents a MaphGV ORF homolog found in other baculoviruses. The red boxes indicate the lepidopteran baculovirus collinearly conserved regions.

Gene content mapping revealed the patterns of baculovirus evolution and highlighted the fluid nature of baculovirus genomes [48]. The gene order of the MaphGV genome was compared with other related betabaculoviruses and representative baculoviruses, including AcMNPV (*Alphabaculovirus, α*), *Neodiprion sertifer nucleopolyhedrovirus* (NeseNPV, *Gammabaculovirus*, γ), and *Culex nigripalpus nucleopolyhedrovirus* (CuniNPV, *Deltabaculovirous*, δ) using the gene parity plot. Gene parity plots allow for comparison of gene organization between two different genomes and are used to illustrate collinearity among baculovirus genomes [25]. The gene order of MaphGV was compared with those of CpGV, CrleGV, PrGV, ChocGV, DisaGV, AcMNPV, NeseNPV, and CuniNPV. The whole-genome nucleotide identity of the MaphGV genome to that of CpGV, CrleGV, PrGV, and DisaGV was 53.8%, 54.8%, 56.8%, and 49.5%, respectively. However, MaphGV shares highly collinear gene order with CrleGV, CpGV, PrGV, and ChocGV, and partial collinearity with AcMNPV (α) (Figure 4). In contrast, its gene arrangement is significantly different from that of NeseNPV (γ) and CuniNPV (δ) (Figure 4). Consistent with previous studies [49], *Betabaculovirus* shares a conserved collinear gene arrangement. Compared with AcMNPV, the genome region between *p48* and *pif-6* is still collinearly conserved (Figure 4).

### 3.4. Repeat Sequences

*hrs* may participate in replication origin of NPVs and GVs and function as enhancers of RNA-polymerase-II-mediated transcription of baculovirus early promoters in NPVs. An individual NPV *hr* typically comprises of a 60–80 bp repeat unit, centered around a palindrome. Unlike NPVs, *hrs* in GVs are more variable and often lack palindromes [49]. For *Betabaculovirus*, conserved *hrs* were found in CpGV, CrleGV, AdorGV, ChocGV, Tortricidae hosts, and PhopGV (Gelechiidae host) with 13 bp imperfect palindrome ends [49]. Although MaphGV is related to CpGV and CrleGV, no typical *hrs* were found.

**Figure 5:**
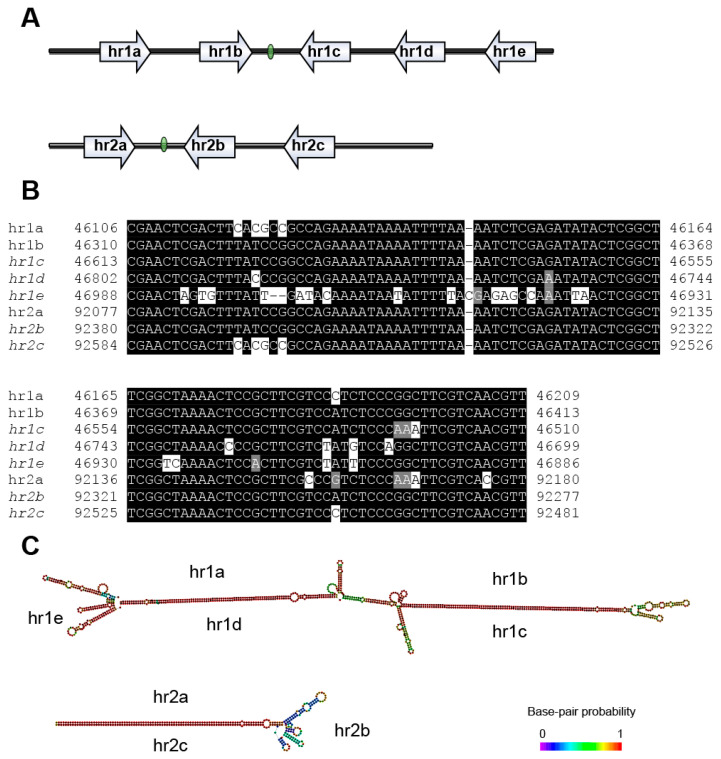
The structure and alignment of MaphGV homologous repeat regions (*hrs*). (**A**) The schematic diagram for the two *hrs* in the MaphGV genome. The blank arrows indicate the location and direction of the *hr* repeat units and the green bead-shaped region indicates the palindromic core. (**B**) Alignment of MaphGV *hr* repeat units. Nucleotide positions of the repeat units in the genome sequence are indicated at both ends of the aligned sequences. The name of the *hr* repeat units occurring on the reverse strand (*hr1c–e*, *hr2b–c*) is indicated in italics beside the alignment. The residues identical to the column-consensus (>50% identical) are shaded in black, whereas gray indicates nucleotides of the same class (containing either a purine or pyrimidine base). (**C**) Predicted secondary structures of MaphGV *hrs*. Secondary structures of single-strand *hrs* DNA were predicted using the ViennaRNA server. The base-pair probabilities of the predicted secondary structure are shown in color.

As a betabaculovirus for tortricids, MaphGV contains two *hrs*, but their structures are atypical (Figure 5A). *hr1* was found in the intergenic region between *sod* and *p74*, with five highly conserved repeat units. *hr2* is located between desmoplakin and *lef-3* and is composed of three repeat units. There is a hypothetical ORF (*orf101*) located in *hr2*. However, no homolog in *Baculoviridae* was found for *orf101*. The repeat units of *hr* are highly conserved within MaphGV, and much longer than typical GV *hr* repeats (104 bp vs. 56–65 bp, Figure 5B). The BLASTn search for the *hr* repeat unit did not reveal any significant hits within an E-value <0.01. In MyunGV [50] and XecnGV-α4 [51], some of the *hr* units are directly repeated, and the other repeat units occur in the reverse orientation, thus forming an imperfect palindrome. Both *hrs* in MaphGV harbor a 12-bp palindromic core GTAAACGTTTAC between the opposite direction repeat units. They likely occur in equilibrium between the double-stranded DNA and opposite hairpin-loops (*hr1*, −504.14 kcal/mol and *hr2*, −201.18 kcal/mol, Figure 5C) constituted by each complementary strand, thus forming a cruciform-like structure. *hr1* and *hr2* are conserved in the MaphGV genome based on positions relative to conserved ORFs [52].

Intergenic repeat sequence analysis also identified four *drs*. The four *drs* were AT-rich, located in distinct intergenic regions (*pep-2*-*vp80*, *vp80*-*orf25*, *orf58*-*orf59*, *orf88*-*orf89*) with different repeat units (Table S4).

One to 16 copies of the baculovirus repeat ORF (*bro*) are present in some betabaculoviruses and all sequenced alpha- and deltabaculoviruses [53]. *bro* comprises a highly repetitive and conserved gene family whose function is unclear [53]. In this study, only one *bro* gene (*orf26*) was found in the MaphGV genome.

### 3.5. iap-6 Found in MaphGV Genome

**Figure 6:**
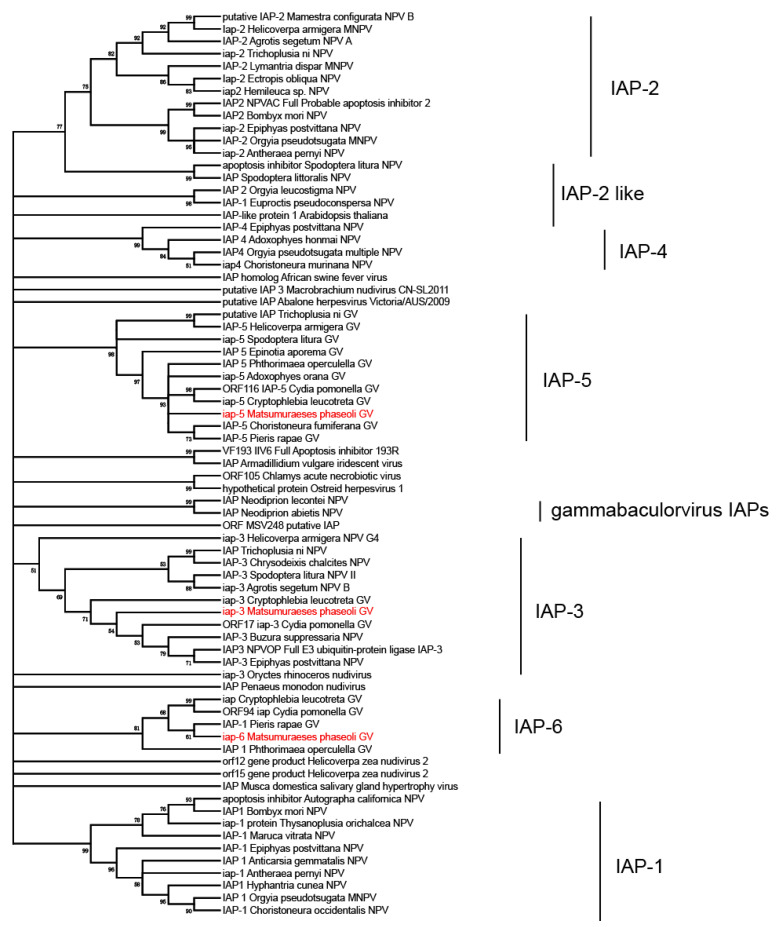
Phylogenetic analysis of the selected viral IAPs. The phylogenetic tree was constructed using the Maximum Likelihood method, using selected baculovirus IAP amino acid sequences with 500 bootstrap values. The initial tree for the heuristic search was obtained by applying the neighbor-joining method to a matrix of pairwise distances estimated using a JTT model. The sequences of IAPs are provided in Table S5. There were 1263 positions in the final dataset. The branches representing MaphGV IAPs are marked in red.

MaphGV contains three *iap* lineages. The *iap* gene is recognized as a central player in regulating apoptosis and many other important processes [54]. IAPs always display at least one baculoviral IAP repeat (BIR) domain, which mediates protein interaction. Baculovirus IAPs also contain a copy of a really interesting new gene (RING) domain in the C-terminus [54]. Alphabaculoviruses carry various combinations of *iap-1*, *iap-2*, *iap-3*, and *iap-4*, whereas most, if not all, betabaculoviruses sequenced to date contain *iap-3* and *iap-5*. Three members of *Gammabaculovirus* carry single *iap* homologs each that are most similar to *iap-3*, but encode only a single BIR and RING, or have two BIRs and lack a RING. Recently, the *iap-6* gene was identified in CpGV, CrleGV, PrGV, and PhopGV, all of which belong to *Betabaculovirus* [55]. In this study, we found not only *iap-3* (*orf11*) and *iap-5* (*orf105*) in the MaphGV genome, but also one *iap-6* (*orf85*). According to phylogenetic analysis, *iap* homologs in MaphGV are well separated, while *iap-6* from MaphGV is clustered with four other *iap-6* from CpGV, CrleGV, PrGV, and PhopGV (Figure 6). These GVs contain *iap-6* grouped in clade b of *Betabaculovirus* (Figure 3). To some extent, the phylogenies of *iap-6* and baculovirus genomes are coherent. Although the phylogeny of the five GVs containing *iap-6* is not strictly monophyletic, it is likely that the *iap-6* homolog descended from a common viral ancestor.

**Figure 7:**
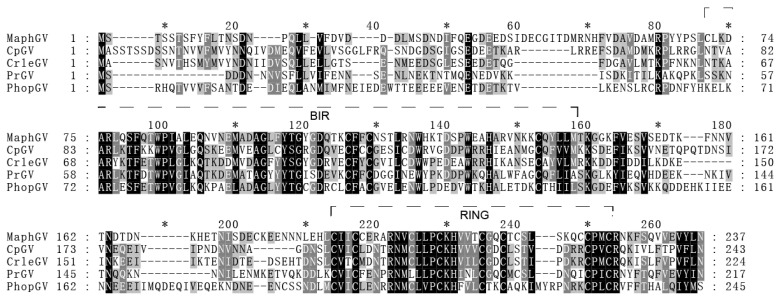
Predicted amino acid sequence alignment of IAP-6. The IAP-6 sequences of *Phthorimaea operculella granulovirus* (NP663251.1), CrleGV (NP891935.1), PrGV (YP003429403.1), CpGV (AIU37301.1), and MaphGV (this study) were aligned using MAFFT with default parameters. The identical amino acids are presented in white with a dark background, and the conserved ones are presented with a grey background. The BIR (71-142 aa) and RING (188-224 aa) domains of MaphGV *iap-6* are indicated by the dashed lines.

MaphGV *orf85* harbors one predicted BIR motif and a C-terminal RING domain, representing highly amino acid conservative property to other *iap-6* (Figure 7) [54]. Considering the phylogenetic relation and domain architecture, we inferred *orf85* of the MaphGV genome as the fifth *iap-6* in *Baculoviridae*. *iap-6* presumably lost a BIR domain during its evolutionary history [54]. Based on the above species phylogenetic analysis, viruses harboring *iap-6* were once closely related. The loss of the BIR domain may have occurred in their common ancestor.

## 4. Conclusions

In this work, we described the genome of a baculovirus isolated from *M. phaseoli*. The virus, named MaphGV, is a novel species, phylogenetically clustered into clade b of the genus *Betabaculovirus*. The virus harbors 128 ORFs, of which 38 are baculovirus core genes, 18 are lepidopteran baculovirus conserved genes, and only 5 are unique to the family *Baculoviridae*. MaphGV is closely related to CpGV and CrleGV, displaying high collinearity with these related species. Two atypical *hrs* were found to be likely form long hairpin-loop structures. Sequencing and profiling of the MaphGV genome may be fundamental in bioinsecticides development. Thus, the discovery and genomic description of the novel baculovirus may guide the development of greener and safer pesticides to counteract and effectively control crop pest populations. These findings have enhanced our understanding on the evolution of baculovirus from a broader perspective.

## Figures and Tables

**Table 1:** Gene contents of MaphGV.

Gene Type	Core Genes	Lepidoptera Baculovirus Conserved Genes	Other Baculovirus Genes
Structure	*odv-e18 (orf13), 49k (orf14), odv-ec43 (orf47), p48/p45 (orf74), p40;bv/odv-c42 (orf76), p6.9 (orf77), 38k (orf79), odv-e25 (orf82), p18 (orf83), p33 (orf84), vp39 (orf87), odv-ec27 (orf88), ac81 (orf93), gp41 (orf94), ac78 (orf95), desmop (orf100), ac53 (orf120), vp1054 (orf123)*	*granulin (orf1), pk-1 (orf3), calyx/pep-1 (orf20), F protein (orf27), bv-e31 (orf61), p24 (orf62), p12 (orf75), tlp-20 (orf92), fp25k (orf118)*	*p10 (orf17), pep-2 (orf23), vp80 (orf24)*
Replication	*lef-2 (orf34), lef-1 (orf64), helicase (orf81), dna-pol (orf99), alk-exo (orf114)*	*lef-11 (orf51), dbp (orf71), lef-3 (orf102), me53 (orf128)*	*ie-1 (orf6), dna ligase (orf108), helicase-2 (orf115)*
Transcription	*p47 (orf60), lef-5 (orf78), lef-4 (orf86), vlf1 (orf96), lef-9 (orf106), lef-8 (orf117)*	*39k (orf50), lef-6 (orf70)*	
Oral infection	*pif-5 (orf16), pif-3 (orf30), pif-2 (orf41), ac110 (orf45), p74 (orf53), pif-1 (orf65), pif-4 (orf80), pif-8 (orf91), pif-6 (orf103)*		*odv-e66 (orf31)*
Auxiliary gene		*38.7k (orf63)*	*chitinase (orf9), iap-3 (orf11), gp37 (orf12), bro (orf26), p13 (orf39), ubiquitin (orf46), sod (orf52), fgf-1 (orf66), iap-1 (orf85), iap-2 (orf105), fgf-2 (orf111), fgf-3 (orf126), egt (orf127)*
Unique gene	*orf22, orf40, orf56, orf101, orf124*		

## Supplementary Materials

The following are available online at https://www.mdpi.com/1999-4915/12/10/1068/s1, Figure S1: SNP of the sequenced MaphGV isolate, Table S1: Primers used in this study, Table S2: Core genes of 108 sequenced baculovirus genomes, Table S3: K2P pairwise distances of selected GVs, Table S4: *drs* in MaphGV, Table S5: *iap* genes of selected species.
