# Supplementary figures and images for: Genome Analysis of a Novel Clade b Betabaculovirus Isolated from the Legume Pest Matsumuraeses phaseoli (Lepidoptera: Tortricidae)
Source: Viruses. 2020 Sep 23;12(10):1068. doi: 10.3390/v12101068 (PMC7650775; doi:10.3390/v12101068)

# SNP of the sequenced MaphGV isolate

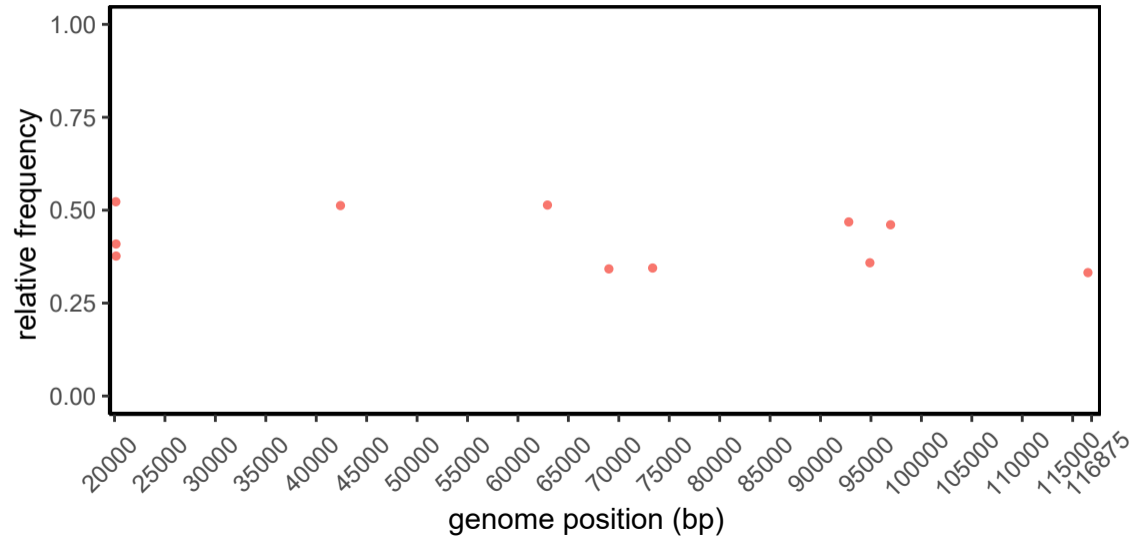

Supplement: Supplementary file 1 [file viruses-12-01068-s001.zip › FigureS1_SNP_of_the_sequenced_MaphGV_isolate.pdf]
